# Impact of co‐morbidities on health‐related quality of life 10 years after surgical treatment of oesophageal cancer

**DOI:** 10.1002/bjs5.50303

**Published:** 2020-05-29

**Authors:** F. Klevebro, A. Johar, P. Lagergren

**Affiliations:** ^1^ Surgical Care Science, Department of Molecular Medicine and Surgery, Karolinska Institutet Karolinska University Hospital Stockholm Sweden; ^2^ Department of Surgery and Cancer Imperial College London London UK

## Abstract

**Background:**

Oesophagectomy for cancer is associated with long‐term decreased health‐related quality of life (HRQoL). The aim of this study was to evaluate the effect of co‐morbidities on HRQoL among survivors of oesophageal or gastro‐oesophageal junctional cancers after 10 years or more.

**Methods:**

The study included a prospectively collected, population‐based cohort, comprising all patients who had surgery for oesophageal or gastro‐oesophageal junctional cancer in Sweden in 2001–2005 with follow‐up until 31 December 2016. All data regarding patient and tumour characteristics, treatment details and HRQoL were collected using a prospectively created database. Multivariable ANCOVA regression models, adjusting for age, sex, tumour histology, stage and surgical technique, were used to calculate adjusted mean scores with 95 per cent confidence intervals for all HRQoL outcomes.

**Results:**

A total of 92 survivors (88·5 per cent) responded to the questionnaires. Patients were stratified in two groups according to whether they reported a low or high impact of co‐morbidities on general health. Patients in the high‐impact group had clinically significantly decreased HRQoL and an increased level of symptoms, but differences between these two groups were not statistically significant.

**Conclusion:**

Co‐morbidities with high impact on general health still contribute to impaired HRQoL 10 years after oesophagectomy for cancer.

## Graphical abstract

This study investigated the impact of co‐morbidities on health‐related quality of life during long‐term follow‐up after oesophagectomy. The results showed that the substantial impact of co‐morbidities on reported general health might be associated with symptoms and health‐related quality of life 10 years after oesophageal cancer treatment.

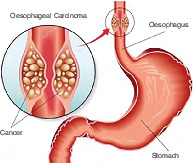

Co‐morbidities affect long‐term QOL.

## Abstract (es)

**Antecedentes:**

La esofaguectomía por cáncer se asocia con un descenso de la calidad de vida relacionada con la salud (*health‐related quality of life*, HRQoL) a largo plazo. El objetivo de este estudio fue evaluar el efecto de las comorbilidades sobre la HRQOL entre pacientes supervivientes de cánceres de esófago o de la unión gastroesofágicas después de 10 años o más.

**Métodos:**

Este estudio incluye una cohorte de base poblacional recogida de forma prospectiva que incluía todos los pacientes operados de cáncer de esófago o de la unión gastroesofágica en Suecia en 2001‐2005 con seguimiento hasta el 31 de diciembre de 2016. Todos los datos relacionados con las características de los pacientes y del tumor, detalles del tratamiento y HRQoL se recogieron en una base de datos prospectiva. Se utilizaron modelos de regresión multivariable ANCOVA, ajustados por edad, sexo, histología del tumor, estadio, y técnica quirúrgica, para calcular las puntuaciones medias ajustadas con los i.c. del 95% para todas las variables de la HRQoL.

**Resultados:**

Un total de 92 (88%) supervivientes respondieron a los cuestionarios. En función del impacto de las comorbilidades en la salud en general, se clasificaron a los pacientes en los grupos de bajo *versus* alto impacto. Los resultados muestran que los pacientes en el grupo de alto impacto presentaban un descenso clínicamente significativo de la HRQoL y un aumento en el nivel de síntomas, pero las diferencias entre estos dos grupos no fueron estadísticamente significativas.

**Conclusión:**

A los 10 años de la esofaguectomía por cáncer, las comorbilidades con un alto impacto sobre la salud general siguen contribuyendo en el deterioro de la HRQoL.

## Introduction

The morbidity of oesophagectomy is among the highest of all surgical procedures^1^ and health‐related quality of life (HRQoL) is affected negatively for up to 10 years after treatment^2^, ^3^. Treatment of oesophageal cancer has improved in recent years, with the introduction of enhanced recovery programmes and minimally invasive surgical techniques^4^, ^5^, but patient characteristics still play an important role. Co‐morbidity at surgery is a known risk factor for poor HRQoL, and acquired co‐morbidities might affect HRQoL in the long term^6^. It is unclear, however, how co‐morbidities are related to long‐term HRQoL after oesophagectomy. The present study aimed to determine whether a high impact of co‐morbidities on subjective general health was associated with decreased HRQoL in patients who had survived 10 years after treatment for oesophageal or gastro‐oesophageal junctional cancer. This might guide tailored follow‐up and influence survivorship.

## Methods

The design of this Swedish nationwide cohort study has been presented in detail elsewhere^7^, ^8^, ^9^, ^10^. The study cohort included 90 per cent of all patients who underwent oesophagectomy for oesophageal or gastro‐oesophageal junctional cancer in Sweden between 1 April 2001 and 31 December 2005, with follow‐up until 31 December 2016. The majority of patients had oesophagectomy alone; 5 per cent received neoadjuvant therapy. Clinical data were collected in a prospectively developed database from medical records based on a predefined study protocol, and included patient and tumour characteristics as well as treatment details.

### Exposure

The study cohort was stratified by the reported effect of co‐morbidity on general health in a follow‐up questionnaire 10 years after treatment. Patients were asked if a physician had diagnosed any co‐morbidities after their oesophageal cancer diagnosis and, if so, to list them and grade the impact of each co‐morbidity on their general health. Possible responses were: 1, not at all; 2, a little; 3, quite a bit; and 4, very much. Patients were stratified in two groups according to the responses: patients without co‐morbidities and those responding 1 or 2 were classified as the low‐impact group; patients with responses 3 or 4 were classified as the high‐impact group.

### Outcomes and clinical data

The outcomes were HRQoL and symptoms reported on the European Organisation for Research and Treatment of Cancer (EORTC) QLQ‐C30 and EORTC QLQ‐OES18 questionnaires. The response alternatives to each question in both questionnaires were: 1, not at all; 2, a little; 3, quite a bit; and 4, very much. The only exception was for the global HRQoL scale; this has two items – self‐reported health and quality of life – with ratings ranging from 1 (very poor) to 7 (excellent). The validated core questionnaire (EORTC QLQ‐C30) was used to measure aspects of HRQoL and symptoms that are applicable to patients with cancer in general, whereas the validated oesophageal cancer‐specific module (EORTC QLQ‐OES18) measured common symptoms among patients with oesophageal cancer. HRQoL was assessed 6 months, 3, 5 and 10 years after surgery. For the purpose of this study, only the 10‐year follow‐up questionnaires were used to compare the two groups. All questionnaires were self‐administered, were delivered by mail and up to three reminders were sent if required. Collection of HRQoL data was done anonymously; patients sent their answers to a central administration and not to the treating department. The Regional Ethical Review Board in Stockholm, Sweden, approved that the study met the requirements for protection of human subjects.

### Statistical analysis

All questionnaire responses were transformed linearly to scores ranging from 0 to 100, according to the scoring procedure in the EORTC manual^11^. Concerning HRQoL, higher scores represent better HRQoL, whereas higher scores in symptom scales represent more symptoms. Missing data were managed as recommended in the EORTC scoring manual^11^. ANCOVA regression models were used to calculate adjusted mean scores with 95 per cent confidence intervals for all HRQoL outcomes. A mean score difference of 10 or more was considered clinically relevant, and was further tested for statistical significance^12^, ^13^, ^14^. The following potential confounders were included in the adjusted models: age (continuous), sex, histological tumour type (squamous cell carcinoma or adenocarcinoma), tumour stage (TNM 0–I, II, III or IV) and surgical technique (transthoracic oesophagectomy, transhiatal oesophagectomy, gastrectomy, or gastrectomy and oesophageal resection combined). All analyses were conducted by an experienced biostatistician according to a predefined study protocol. The statistical software used was SAS® version 9.4 (SAS Institute, Cary, North Carolina, USA).

## Results

Of 616 patients included in the original study, 104 (16·9 per cent) were alive, of whom 92 (88·5 per cent) responded to HRQoL questionnaires 10 years after treatment. The impact of co‐morbidities on general health was reported as none or low by 60 patients (65 per cent), whereas 32 patients (35 per cent) reported a high impact. Patient characteristics were similar in the low‐ and high‐impact groups (*Table* *1*).

**Table 1:** Characteristics of patients included in the health‐related quality‐of‐life analysis 10 years after treatment with curative intent for oesophageal or gastro‐oesophageal junctional cancer

	Low impact of co‐morbidity (*n* = 60)	High impact of co‐morbidity (*n* = 32)
Age (years)*	73 (41–89)	73 (58–84)
**Sex ratio (F** : **M)**	14 : 46	5 : 27
**Histological tumour type**		
Squamous cell carcinoma	12 (20)	8 (25)
Adenocarcinoma	48 (80)	24 (75)
**Tumour stage**		
0–I	32 (53)	17 (53)
II	17 (28)	12 (38)
III	10 (17)	3 (9)
IV	1 (2)	0 (0)
**Surgical approach**		
Transthoracic oesophagectomy	51 (85)	25 (78)
Transhiatal oesophagectomy	8 (13)	6 (19)
Three‐stage oesophagectomy	1 (2)	1 (3)

Values in parentheses are percentages unless indicated otherwise; *values are mean (range).

The 60 patients in the low‐impact group reported a total of 47 co‐morbidities, and the 32 patients in the high‐impact group reported 51 (*Table* *2*). Gastrointestinal co‐morbidities were the most common in both groups.

**Table 2:** Co‐morbidities listed by patients 10 years after treatment with curative intent for oesophageal or gastro‐oesophageal junctional cancer

	Low impact of co‐morbidity (*n* = 60)	High impact of co‐morbidity (*n* = 32)
Gastrointestinal	11 (18)	8 (25)
Cardiovascular	9 (15)	7 (22)
Pulmonary	3 (5)	6 (19)
Malignancy	9 (15)	7 (22)
Diabetes	0 (0)	1 (3)
Orthopaedic	1 (2)	6 (19)
Urological	7 (12)	7 (22)
Neurological	0 (0)	1 (3)
Autoimmune	0 (0)	2 (6)
Other	7 (12)	6 (19)
		
Total no. of co‐morbidities*	47	51

*Each patient could report more than one co‐morbidity.

Patients who experienced a high impact of co‐morbidity on health reported clinically relevant worse scores for role and social functioning, as well as financial difficulties, but the differences were not statistically significant (*Table* *3*). Similarly, patients in the high‐impact group reported more pain, appetite loss and diarrhoea considered clinically relevant, but this was not statistically significant. With regard to oesophageal‐specific symptoms, clinically more problems were reported for eating issues and dry mouth in the high‐impact group than in the low‐impact group, but again without statistical significance (*Table* *3*). Insomnia and fatigue were reported at relatively high levels in both groups.

**Table 3:** Health‐related quality of life 10 years after oesophageal cancer surgery assessed using EORTC questionnaires, according to impact of co‐morbidity

	Adjusted mean score*	
	Low impact of co‐morbidity (*n* = 60)	High impact of co‐morbidity (*n* = 32)
**EORTC QLQ‐C30**		
Global health status	69·5 (52·9, 86·1)	63·2 (45·5, 81·0)
Physical functioning	81·7 (66·5, 96·8)	78·8 (62·7, 94·9)
Role functioning	75·6 (51·6, 99·5)	65·7 (39·8, 91·5)†
Emotional functioning	86·9 (70·8, 102·9)	81·8 (64·7, 100·0)
Cognitive functioning	91·6 (75·4, 107·7)	90·8 (73·5, 108·0)
Social functioning	93·0 (75·6, 110·4)	78·8 (60·2, 97·4)†
Fatigue	25·3 (4·9, 45·7)	26·1 (4·3, 47·9)
Nausea and vomiting	13·5 (–5·7, 15·4)	17·4 (–3·1, 37·9)
Pain	10·5 (–8·6, 29·5)	21·8 (1·4, 42·2)†
Dyspnoea	17·5 (–5·9, 41·0)	22·2 (–2·9, 47·3)
Insomnia	29·2 (4·8, 53·5)	25·0 (–1·1, 51·0)
Appetite loss	12·3 (–14·9, 39·5)	22·4 (–6·7, 51·6)†
Constipation	5·7 (–12·4, 23·7)	9·9 (–9·4, 29·1)
Diarrhoea	25·3 (3·4, 47·1)	37·7 (14·5, 60·9)†
Financial difficulties	2·4 (–15·1, 19·8)	14·4 (–4·3, 33·2)†
**EORTC QLQ‐OES18**		
Dysphagia	30·0 (10·4, 49·6)	21·4 (0·3, 42·6)
Eating problems	28·2 (9·8, 46·6)	38·9 (19·2, 58·6)†
Reflux	34·7 (13·4, 56·1)	34·5 (11·7, 57·4)
Pain	13·9 (–2·5, 30·2)	21·9 (4·4, 39·4)
Trouble swallowing saliva	12·0 (–2·8, 26·8)	3·6 (–13·5, 20·7)
Choked when swallowing	11·5 (–8·0, 31·1)	16·6 (–4·5, 37·7)
Dry mouth	7·8 (–12·2, 27·8)	22·3 (0·9, 43·7)†
Trouble with taste	12·8 (–6·4, 31·9)	16·6 (–3·8, 37·0)
Trouble with coughing	10·2 (–12·9, 33·4)	11·4 (–13·3, 36·2)
Trouble with talking	18·6 (2·5, 34·7)	23·8 (6·6, 41·0)

Values in parentheses are 95 per cent confidence intervals. *Adjusted for age, sex, histological tumour type, tumour stage and surgical technique. Model for trouble swallowing saliva was adjusted only for age and stage; other confounders were removed owing to issues with model estimation. †Clinically significant differences. EORTC, European Organisation for Research and Treatment of Cancer.

## Discussion

This study showed that co‐morbidity affecting patients' reported general health to a high level had a negative impact on role and social functioning, and increased financial difficulties, pain, appetite loss and diarrhoea 10 years after oesophageal cancer surgery. Although differences were not statistically significant, they reached a level of clinical importance. These results provide some explanation as to why some patients still report poor HRQoL 10 years after cancer treatment.

Strengths of the study include the population‐based prospective design, and long follow‐up. HRQoL was measured using validated questionnaires, which reduces the risk of information bias and increases the comparability of the results with those of other research studies. All clinical data were collected according to a predefined protocol, and medical records were scrutinized by researchers not involved in the care of the patients. The variables included in the multivariable regression model were prespecified. Limitations of the study include the small sample size, which reflects the poor long‐term survival of patients with oesophageal cancer. The response rate among surviving patients was very high, which reduces the risk of selection bias.

In a previous study^3^ based on the same cohort, HRQoL among patients 10 years after treatment was reported as decreased in all measured variables compared with a matched reference population; the results also showed a decline in 23 of the 25 variables measured between 5 and 10 years after operation. The reasons behind this deterioration in surviving patients are likely to be multifactorial. Other studies^15^, ^16^, ^17^ have shown that complications of treatment as well as eating problems have a significant impact on HRQoL 10 years after treatment. In an analysis of HRQoL in the present cohort 5 years after treatment^6^, patients with increased co‐morbidities showed a deterioration in HRQoL compared with those who had no or stable co‐morbidities, indicating that co‐morbidities have an important impact on long‐term HRQoL.

Co‐morbidities with a high reported impact on general health can be associated with symptoms and decreased HRQoL for more than 10 years after oesophagectomy for cancer. This information may be useful in tailoring the follow‐up and influence survivorship.

## References

[1] Schieman C , Wigle DA , Deschamps C , Nichols FC III , Cassivi SD , Shen KR *et al* Patterns of operative mortality following esophagectomy. Dis Esophagus 2012; 25: 645–651. 22243561 10.1111/j.1442-2050.2011.01304.x

[2] Scarpa M , Valente S , Alfieri R , Cagol M , Diamantis G , Ancona E *et al* Systematic review of health‐related quality of life after esophagectomy for esophageal cancer. World J Gastroenterol 2011; 17: 4660–4674. 22180708 10.3748/wjg.v17.i42.4660 PMC3233672

[3] Schandl A , Lagergren J , Johar A , Lagergren P . Health‐related quality of life 10 years after oesophageal cancer surgery. Eur J Cancer 2016; 69: 43–50. 27816831 10.1016/j.ejca.2016.09.032

[4] Wang L , Zhu C , Ma X , Shen K , Li H , Hu Y *et al* Impact of enhanced recovery program on patients with esophageal cancer in comparison with traditional care. Support Care Cancer 2017; 25: 381–389. 27726032 10.1007/s00520-016-3410-0

[5] Gemmill EH , Humes DJ , Catton JA . Systematic review of enhanced recovery after gastro‐oesophageal cancer surgery. Ann R Coll Surg Engl 2015; 97: 173–179. 26263799 10.1308/003588414X14055925061630 PMC4474007

[6] Backemar L , Wikman A , Djarv T , Johar A , Lagergren P . Co‐morbidity after oesophageal cancer surgery and recovery of health‐related quality of life. Br J Surg 2016; 103: 1665–1675. 27545978 10.1002/bjs.10248

[7] Derogar M , Lagergren P . Health‐related quality of life among 5‐year survivors of esophageal cancer surgery: a prospective population‐based study. J Clin Oncol 2012; 30: 413–418. 22215745 10.1200/JCO.2011.38.9791

[8] Viklund P , Lindblad M , Lu M , Ye W , Johansson J , Lagergren J . Risk factors for complications after esophageal cancer resection: a prospective population‐based study in Sweden. Ann Surg 2006; 243: 204–211. 16432353 10.1097/01.sla.0000197698.17794.eb PMC1448902

[9] Wikman A , Ljung R , Johar A , Hellstadius Y , Lagergren J , Lagergren P . Psychiatric morbidity and survival after surgery for esophageal cancer: a population‐based cohort study. J Clin Oncol 2015; 33: 448–454. 25547500 10.1200/JCO.2014.57.1893

[10] Djarv T , Blazeby JM , Lagergren P . Predictors of postoperative quality of life after esophagectomy for cancer. J Clin Oncol 2009; 27: 1963–1968. 19289614 10.1200/JCO.2008.20.5864

[11] Fayers PAN , Bjordal K , Groenvold M , Curran D , Bottomley A . The EORTC QLQ‐C30 Scoring Manual. European Organisation for Research and Treatment of Cancer: Brussels, 2001.

[12] Osoba D , Rodrigues G , Myles J , Zee B , Pater J . Interpreting the significance of changes in health‐related quality‐of‐life scores. J Clin Oncol 1998; 16: 139–144. 9440735 10.1200/JCO.1998.16.1.139

[13] Blazeby JM , Alderson D , Winstone K , Steyn R , Hammerlid E , Arraras J *et al* Development of an EORTC questionnaire module to be used in quality of life assessment for patients with oesophageal cancer. The EORTC Quality of Life Study Group. Eur J Cancer 1996; 32A: 1912–1917. 8943674 10.1016/0959-8049(96)00199-2

[14] Aaronson NK , Ahmedzai S , Bergman B , Bullinger M , Cull A , Duez NJ *et al* The European Organization for Research and Treatment of Cancer QLQ‐C30: a quality‐of‐life instrument for use in international clinical trials in oncology. J Natl Cancer Inst 1993; 85: 365–376. 8433390 10.1093/jnci/85.5.365

[15] Kauppila JH , Johar A , Lagergren P . Medical and surgical complications and health‐related quality of life after esophageal cancer surgery. Ann Surg 2018; 271: 502–508. 10.1097/SLA.0000000000003097 30339626

[16] Kauppila JH , Johar A , Lagergren P . Postoperative complications and health‐related quality of life 10 years after esophageal cancer surgery. Ann Surg 2018; 271: 311–316. 10.1097/SLA.0000000000002972 29995688

[17] Anandavadivelan P , Wikman A , Johar A , Lagergren P . Impact of weight loss and eating difficulties on health‐related quality of life up to 10 years after oesophagectomy for cancer. Br J Surg 2017; 105: 410–418. 29160918 10.1002/bjs.10686

